# Effectiveness of anti-inflammatory treatment versus antibiotic therapy and placebo for patients with non-complicated acute bronchitis with purulent sputum. The BAAP Study protocol

**DOI:** 10.1186/1471-2466-11-38

**Published:** 2011-06-21

**Authors:** Carl Llor, Ana Moragas, Carolina Bayona, Rosa Morros, Helena Pera, Josep M Cots, Yvonne Fernández, Marc Miravitlles, Albert Boada

**Affiliations:** 1 University Rovira i Virgili. Primary Healthcare Centre Jaume I, Tarragona, Spain; 2 Primary Healthcare Centre Jaume I, Tarragona, Spain; 3 Primary Healthcare Centre Valls Urbà, Valls (Tarragona), Spain; 4 Fundació d'Investigació d'Atenció Primaria Jordi Gol i Gurina, Spain; 5 University of Barcelona. Primary Healthcare Centre La Marina, Barcelona, Spain; 6 Department of Pneumology. Institut Clínic del Tòrax (IDIBAPS), Hospital Clínic, Barcelona. CIBER de Enfermedades Respiratorias (CIBERES), Spain; 7 Primary Healthcare Centre Maragall, Barcelona, Spain

## Abstract

**Background:**

Acute bronchitis is one of the most prevalent respiratory infections in primary care, and in more than 90% of the cases antibiotics are prescribed, mainly when purulent expectoration is present. However, this process is usually viral in origin and the benefits of antibiotic treatment are marginal. On the other hand, in recent years bronchitis has been considered more as an inflammatory than an infectious process. Thus, the aim of this study is to evaluate the clinical effectiveness of a schedule of an oral anti-inflammatory compared with an antibiotic regimen and another group assigned to receive a placebo.

**Methods and design:**

A total of 420 patients from 15 to 70 years of age with no associated comorbidity, presenting respiratory tract infection of at least one week of evolution, with cough as the predominant symptom, the presence of purulent expectoration and at least one other symptom of the respiratory tract (dyspnoea, wheezing, chest discomfort or pain), with no alternative explanation such as pneumonia, will be included in a prospective, randomised and controlled, clinical trial with placebo. The patients will be randomised to receive one of three treatments: ibuprofen, amoxycillin and clavulanic acid or placebo for 10 days. The main outcome measure is the number of days with frequent cough defined by the symptom diary with a score of 1 or more.

**Discussion:**

This trial is designed to evaluate the number of days with frequent cough with anti-inflammatory treatment compared with antimicrobial treatment and placebo in previously healthy patients with a clinical picture of acute bronchitis and purulent expectoration. It is hypothesized that anti-inflammatory treatment is more effective than antibiotic treatment to reduce cough, which is the most disturbing symptom for patients with this infection.

**Trial registration:**

ISRCTN07852892

## Background

Acute bronchitis is a clinical term which implies a self-limiting infection of the large airways and is characterized by clinical manifestations of cough without pneumonia [1]. This process affects approximately 5% of adults annually with a greater incidence in the winter. It accounts for 9.4% of the infections attended by family physicians [2]. Acute bronchitis is mainly a viral infection [3,4]. The role of bacteria in this infection continues to be controversial since bronchial biopsies have never demonstrated bacterial invasion. In some cases atypical germs may be involved including *Bordetella pertussis, Chlamydophila pneumoniae *and *Mycoplasma pneumonia *[1,5].

It is thought that acute bronchitis reflects an inflammatory response to infections of the epithelium of the bronchi. Microscopic examination demonstrates a thickening of the bronchial and tracheal mucosa corresponding to the inflamed areas. These pathological findings are consistent with publications of proximal lower airway inflammation observed with positron emission tomography [6]. During the first days of infection, the symptoms of upper respiratory infections cannot be clinically distinguished from the symptoms of acute bronchitis. However, in acute bronchitis cough lasts more than five days and during this prolonged period of symptoms, the results of functional respiratory tests are usually abnormal. Forty per cent of the patients present significant reductions in forced espiratory volume in the first second (FEV_1_) with values of less than 80% over the theoretical value, or bronchial hyper-reactivity with improvement during the following 5-6 weeks [7]. Cough may persist from 10 to 20 days in acute bronchitis, but it may sometimes last up to 4 weeks or more. In a recent study on the efficacy of the acellular pertussis vaccine including 2,781 healthy adults, the mean duration of cough in acute bronchitis by all causes was 18 days, with a median of 24 days [5]. Moreover, almost half of the patients with acute bronchitis report the production of purulent expectoration. Peroxidase released by the leukocytes in sputum causes the colour changes; hence, colour alone should not be considered indicative of bacterial infection [8]. Indeed, in healthy adults, the positive predictive value of purulent sputum for the presence of alveolar disease is low, being of approximately 10% [9].

Bronchitis has shown to reduce the quality of life of patients. One study on the quality of life of patients with infections of the upper respiratory tract, some of which were labelled acute bronchitis, showed a reduction in seven subscales of the *36-item Short-Form General Health Survey*, including vital and social function, although this decrease was transitory [10]. Furthermore, up to twenty per cent of the patients return to their physician within the first month for the persistence of symptoms [1].

### Previous research

There is clear evidence that antibiotics should not be recommended in non-complicated acute bronchitis. However, their use on behalf of primary care physicians in Spain is greater than 80% [11,12]. Similar percentages have been reported by primary care practitioners in United States and other countries [13,14]. Systematic analyses of clinical trials suggest that antibiotics may achieve a reduction, albeit only marginal, in the duration of the symptoms. Specifically, a meta-analysis of 8 clinical trials showed that the symptoms reduced a few hours with the use of doxycycline, erythromycin or trimethoprim/sulphamethoxazole [15]. The results were statistically significant but clinically trivial. The results of a randomized, double-blind study in which a course of azithromycin during 5 days in 112 patients was compared with vitamin C in 108 patients published after this meta-analysis did not show any difference between groups in the quality of life at 7 days (main outcome variable) or in the proportion of patients who had to return to work, school or normal activities at home at days 3 or 7 [16]. A review of the *Cochrane Library *including 9 randomized, controlled, clinical trials of antibiotic agents (including three clinical trials not included in the previous review) also showed a significant, albeit lower, reduction in the duration of cough, of only 0.6 hours [17]. A non-significant reduction in the number of days of feeling badly and a non-significant increase in the number of adverse effects attributed to the antibiotic therapy (relative risk of adverse effects: 1.22; CI95%: 0.94 to 1.58) were also found [17]. At this point, the study by Stott *et al. *is of note in which only patients with acute bronchitis with purulent expectoration were included and in which no statistically significant differences were observed in the resolution of cough between the group treated with antibiotics and those assigned to the control group [18]. Thus, Spanish primary care physicians have the perception that the presence of purulent expectoration in the course of acute bronchitis is suggestive of bacterial superinfection, very probably due to the fact that purulent expectoration is a criterion of antibiotic therapy in patients with other lower respiratory tract infections such as acute exacerbations of chronic obstructive pulmonary disease [19]. Indeed, in a questionnaire given to more than 1,000 primary care physicians, more than 90% responded that they would treat a 30-year-old patient with associated comorbidity presenting a non-pneumonic respiratory infection with sputum purulence with antibiotics [20].

In recent years the idea that acute bronchitis is more an inflammatory than infectious process has begun to take shape [1,21]. Thus, non-steroidal anti-inflammatory drugs have shown to be effective in reducing the duration and the intensity of cough in patients with bronchitis in observational studies and they are therefore recommended in the guidelines of clinical practice. However, their effectiveness has not been observed in clinical trials. To date, no study has been published comparing the role of anti-inflammatory drugs with antibiotics. We therefore designed this clinical trial with the aim of determining the effectiveness of ibuprofen (most frequently prescribed anti-inflammatory drug in Spain) compared with the combination of amoxicillin and clavulanic acid (antibiotic most often prescribed by Spanish primary care physicians) and also compared with a placebo groups of healthy adult patients with acute bronchitis and purulent expectoration.

### Objectives

The aim of the study is to evaluate the number of days of frequent cough (defined by the symptom diary with a score of 1 or more) in the three treatment arms (oral anti-inflammatory, antibiotic or placebo) taken during 10 days. The secondary objectives are to evaluate the efficacy on the second visit, evaluate the speed of action in the resolution of the symptoms of acute bronchitis in the three treatment arms, assess the relationship between C-reactive protein concentrations and the resolution of the symptoms in each treatment arm and evaluate the secondary effects and adverse reactions in the three treatment arms of the study.

## Methods

### Design of the study

The study entails a 3-armed randomized clinical trial performed in Catalonia, Spain. The medical Ethics Committee *Fundació d'Investigació en Atenció Primària *has approved the study design, the protocols, information letters to the patients and the informed consent form. The study has obtained a grant from the *Fondo de Investigación Sanitaria *of the Ministry of Health and Consumption in the announcement of independent investigation and has been approved by the Spanish Agency of Medication and Healthcare Products.

### Recruitment

Patients from 18 to 70 years of age without associated respiratory comorbidity or immunosuppression, presenting respiratory infection of at least one week of evolution, with cough as the predominant symptoms and the presence of purulent expectoration of at least one week of duration and at least one other respiratory tract symptom such as dyspnoea, wheezing, chest discomfort or pain, with no alternative explanation such as pneumonic condensation, will be recruited from several healthcare centres in Catalonia.

Exclusion criteria will be: patients less than 18 and older than 70 years of age, the presence of radiological signs of pneumonia, signs of severe infection (confusion, tachypnoea > 25 respirations per minute of tachycardia > 120 beats per minute), history of digestive haemorrhage or intolerance to anti-inflammatory treatment, hypersensitivity to β-lactams or intolerance to clavulanic acid or lactose, pregnancy, lactation and women of fertile age not using contraceptive measures, antibiotic, anti-inflammatory or corticosteroid use in the previous two weeks, associated comorbidity (bronchial asthma, chronic obstructive pulmonary disease, moderate-severe heart failure, dementia, stroke, immunosuppression or the use of immunosuppressive drugs), emergency situation, institutionalization in a residence, and/or subjects unable to personally provide informed consent.

### Randomization

The patients will be randomized with simple random numbers to one of the three following treatment arms:

• Ibuprofen 600 mg/8 hours, during 10 days, taken after meals.

• Amoxicillin plus clavulanic acid 500-125 mg/8 hours, during 10 days, taken after meals.

• Placebo, one tablet every 8 hours, during 10 days, taken after meals.

The use of acetaminophen will be allowed in cases of fever or pain, as will short life β-adrenergics or anticholinergics in cases of bronchospasm and mucolytics, anti-cough and any medication that the patient may be taking for chronic disease and which has been initiated one month prior to inclusion in the study except for antibiotics, inhaled, oral or parenteral corticoids or non-steroidal anti-inflammatory drugs. In cases of clinical deterioration, the medical investigator will evaluate the need for hospitalisation and in the case of outpatient treatment, high dose antibiotic treatment with amoxycillin and clavulanic acid will be administered.

The patient will have to return all the medication samples not taken to the investigator. If the left over medication is not returned, adherence will be assessed as insufficient and the case will be classified as abandonment. Prescription adherence will be evaluated and will be considered satisfactory if the patient has taken at least 80% of the dose foreseen.

### Measurements

The main outcome variable considered in this study is the number of days with frequent cough. Secondary result variables considered will be the efficacy of the treatment, the time of symptom resolution, the reduction in the total daily score of the symptom diary and the secondary effects and adverse reactions which may present with the three treatment arms. With regard to efficacy, three outcomes will be distinguished: cure, defined as the disappearance of the acute signs and symptoms related to the infection (complete return to the previous situation of stability), improvement, defined as the non-complete resolution of the symptoms and failure, with an insufficient reduction in the signs and symptoms of infection.

The following variables will be taken into account in this study:

• Age, sex, presence of smoking, packets-year, presence of diabetes, heart failure, ischemic heart disease.

• Treatment administered by the physician: mucolytics or expectorants, antihistaminics, bronchodilatadors, antitussives, analgesics.

• Colour of the sputum: yellowish or yellowish-green.

• Clinical manifestations of the patient: dyspnoea, wheezing on auscultation, chest discomfort, chest pain, pain (>38°C).

• Concentration of C-reactive protein in capillary blood, with quantitative apparatus (QuikRead of Orion Diagnostica).

• Chest X-ray: will be considered positive if there is any complication such as pulmonary condensation (in which case, the patient will be excluded from the study); or negative.

• Symptom diary: Five symptoms will be evaluated: disease severity, day-time cough, night-time cough, limitation in daily activity and febrile sensation. Each of the symptoms will be scored from 0 to 4. It will be recommended that the patient fill out this diary before going to bed. This symptom diary has previously been used in other studies [22].

• Secondary effects and adverse reaction which might appear in the three treatment arms.

Four visits have been established in this study (Table 1). On the baseline visit the study will be explained to the patient and informed consent will be requested. The scheme of the investigation will also be explained to the patient. The patients will be randomized to any of the three treatment groups and the medication will be given with 33 tablets (three extra tablets are given in case of loss). On this visit the C-reactive protein will be determined and a chest X-ray will be performed in cases with suspected pneumonia. In addition, a symptom diary will be given as will an explanation as to how to fill it in.

**Table 1:** Study measurements

	Baseline	2-4 days	11-13 days	30 days
**Randomization**	x			

**C-reactive protein**	x			

**Chest X-ray**	x			

**Hand-out of the symptom diary**	x		x (1)	

**Side effects and adverse reactions assessment**		x	x	x (1)

**Evaluation of clinical evolution**		x	x	x (1)

**Review of adherence**			x	

**Collection of symptom diary**			x	x (2)

(1) If the patient continues to have symptomatology on day 11-13

(2) If the patient has been given the second diary symptom

The first follow up visit will be programmed at 2-4 days. On this visit the presence of side effects and adverse reactions will be assessed. In addition, the clinical evolution of the symptoms and signs will be evaluated and, in the case of worsening of the symptoms, hospital referral or the administration of antibiotic treatment will be considered. The second follow up visit will be made at 11-13 days with a review of adherence to the study drug and an evaluation of the presence of side effects and adverse reactions. The patients classified as clinical failure will be managed as in the previous visit. The symptom diary will be collected and another diary will be given if the patient continues to have symptomatology. Patients classified as cure will finish the study on this visit and those classified as improvement will be given an appointment at 30 days of having initiated the treatment with a new diary, considering this as the last visit of the study.

The data of the study will be collected on a data collection sheet, a one-sided form sheet which the participating investigator will have identified with the number of randomization indicated on this sheet, on the symptom sheet given to the patient and on the medication bottle given to the patient. The randomization codes will be kept by the Pharmacy Unit of the *Hospital Vall d'Hebron *of Barcelona.

### Sample size

We will accept a null hypothesis if the number of days of frequent cough in the anti-inflammatory arm is the same or ± 2 days as that observed in the other two treatment arms (placebo and antibiotic treatment). Based on the literature, the standard deviation of the duration of frequent cough is 5.5 days in patients with chronic bronchitis and purulent sputum. For an α: 0.05 and a β: 0.2 and accepting possible losses of 15%, we calculate that the sample size should be of 140 patients per group (total of 420 patients).

### Analysis

Intention-to-treat statistical analyses will be performed. To analyse the time until cure of the different symptoms and for the main result variable (days from the onset of the picture until the patient scores a maximum of 1 in the box related to cough in the symptom diary) survival analysis will be carried out using the Kaplan-Meier method. Comparison between the three survival curves will be undertaken using the log-rank test. In addition, bivariate analysis will be peformed with comparison of proportions using the chi-square test to evaluate secondary variables of the study and variance analysis to assess the relationship between quantitative variables for both independent and paired data.

### Ethical considerations

Approval has been obtained from the Ethical Committee of Investigation in Primary Care *(Fundació d'Investigació en Atenció Primària)*. The clinical trial has been registered in an international database (ISRCTN07852892). Informed consent will be requested from all the participants in the study, respecting subject autonomy in their decision. To achieve this, the medical investigator will read the informed consent sheet to the patients and clarify any doubt which might arise during the reading. Thereafter, patient comprehension of the information will be determined and, in the case of acceptation to participate, the patient will be asked to sign the form. This study does not require the use of unusual, additional tests: only a chest X-ray will be undertaken to rule out a pneumonic process and determination of the C-reactive protein during the visit to know the severity of the infection, being a test that is total justified in this project.

From an ethical point of view, this is to certify that the objective of this study is important and relevant for primary care, the power of the study may be considered as reasonable, this is an original study, the risks which the participants may incur justify the investigation being carried out with a totally favourable benefit/risk quotient, and we ensure the external validity of the study to the primary care reality, with the inclusion and exclusion criteria described. Vulnerable populations will not participate in this study and neither will economic compensation be given to patients for their participation. The investigators are free to publish the results of this study, regardless of the results obtained. We guarantee the anonymity and confidentiality of the data, the protocol of the study will safeguard that in results of the study all the participants and their individual results will be taken into account. The privacy of the participants will be protected and with the randomised design of the study the subjects will be equally invited to participate and we consider that the possible benefits or adverse effects of the investigation will be equally shared among all the participants.

## Discussion

This article presents a detailed description of a randomized clinical trial protocol study designed to explore the effectiveness of ibuprofen to alleviate the cough present in patients with acute bronchitis and purulent expectoration compared with a broad-spectrum antibiotic and placebo. The trial proposed is sufficiently powered and, to the best of our knowledge, the first to allow conclusions on the effect of an anti-inflammatory drug and compare it with antibiotic therapy. Acute bronchitis is a very prevalent disease and is one of the most frequent causes of medical visits in primary care. Patients often return to their physician or seek other medical help because the symptoms of the disease may persist for two or three weeks, mainly cough, which may be very bothersome for the patient. Many physicians may not give antibiotics on the first visit and may prescribe mucolytics and/or antitussives. However, since healthcare professionals often do not explain the natural evolution of bronchitis and since the patients are often told that these drugs will cure the patient, the patient returns with the same demand. Butler *et al. *reported similar results, with only 5% of the professionals explaining to their patients with bronchitis that the cough would last at least two weeks [23]. When the patient returns and particularly when the patient presents purulent mucous expectoration, the physician is more likely to prescribe an antibiotic [24]. Thus, only patients with acute bronchitis and yellowish-green expectoration will be included in this clinical trial.

This study will involve the use of drugs which have been in the pharmaceutical market for more than 20 years. Moreover, we will take into account the most commonly used drugs in Spain for each of the two pharmacological groups: ibuprofen among the anti-inflammatory drugs and amoxicillin and clavulanic acid as the antibiotic used in lower respiratory tract infections. This is, therefore, a phase IV clinical trial and, as such, we believe that important adverse effects will not be produced. This trial will deliver important findings on the effects of these drugs and the results will provide information on which of the two drugs produces the best results to alleviate cough using the symptom diary which the patients will be trained to fill in.

## Competing interests

The authors declare that they have no competing interests.

## Authors' contributions

CL, AM, CB, RM, HP, JMC, YF, MM, and AB designed this study protocol. CL and AM drafted the manuscript and CB, RM, HP, JMC, YF, MM, and AB contributed to the manuscript. All authors have read and approved the final manuscript.

## Pre-publication history

The pre-publication history for this paper can be accessed here:

http://www.biomedcentral.com/1471-2466/11/38/prepub
